# Supplementary material for: Human Exposure to Naturally Occurring Bacillus anthracis in the Kars Region of Eastern Türkiye
Source: Microorganisms. 2024 Jan 14;12(1):167. doi: 10.3390/microorganisms12010167 (PMC10818668; doi:10.3390/microorganisms12010167)
Supplement: Supplementary file 1 [file microorganisms-12-00167-s001.zip › microorganisms-2769165-supplementary.pdf]

Human exposure to naturally occurring *Bacillus anthracis* in the Kars region of eastern Türkiye

Supplementary Tables

Table S1: Descriptive statistics of characteristics of study participants

Descriptive statistics are shown for two groups of participants: (1) with known previous anthrax infection, (2) with no history of anthrax infection. Where count data are used, the number of participants is given with the percentage of the parent population. Where data are continuous, the mean (μ) is given with the standard deviation. [n/a = not applicable].

Parameter		Total	Confirmed previous anthrax infection	No history of anthrax infection
Anthrax infection history		$n = 279$	$n = 105\ (37.6\%)$	$n = 174\ (62.4\%)$
Time from diagnosis [y]		n/a	$\mu = 4.6\ (\pm 4.8)$	n/a
Age [y]		$\mu = 38.7\ (\pm 12.2)$	$\mu = 43.9\ (\pm 14.3)$	$\mu = 35.6\ (\pm 9.5)$
Time in at risk occupation [y]		$\mu = 28.2\ (\pm 18.4)$	$\mu = 41.5\ (\pm 16.1)$	$\mu = 20.2\ (\pm 14.7)$
Altitude [m]		$\mu = 1817\ (\pm 122)$	$\mu = 1846\ (\pm 143)$	$\mu = 1800\ (\pm 104)$
Population of home settlement		$\mu = 32K\ (\pm 37K)$	$\mu = 18K\ (\pm 0.1K)$	$\mu = 41K\ (\pm 38K)$
Gender	Male	$n = 236\ (84.6\%)$	$n = 86\ (81.9\%)$	$n = 150\ (86.2\%)$
	Female	$n = 43\ (15.4\%)$	$n = 19\ (18.1\%)$	$n = 24\ (13.8\%)$
Known contact event		$n = 76\ (27.2\%)$	$n = 4\ (3.8\%)$	$n = 72\ (41.4\%)$
Time since contact event [y]		$\mu = 3.6\ (\pm 2.3)$	$\mu = 3.2\ (\pm 0.8)$	$\mu = 3.6\ (\pm 2.4)$
Continuous risk of spore exposure		$n = 11\ (3.9\%)$	$n = 4\ (3.8\%)$	$n = 7\ (4\%)$
Duration of Continuous risk [y]		$\mu = 23.1\ (\pm 8)$	$\mu = 25\ (\pm 12.9)$	$\mu = 22\ (\pm 4.6)$
Occupation group	Butcher	$n = 48\ (17.2\%)$	$n = 13\ (12.4\%)$	$n = 35\ (20.1\%)$
	Lab staff	$n = 12\ (4.3\%)$	$n = 0$	$n = 12\ (6.9\%)$
	Leather worker	$n = 5\ (1.8\%)$	$n = 1\ (1\%)$	$n = 4\ (2.3\%)$
	Shepherd	$n = 29\ (10.4\%)$	$n = 0$	$n = 29\ (16.7\%)$
	Urban dweller	$n = 31\ (11.1\%)$	$n = 13\ (12.4\%)$	$n = 18\ (10.3\%)$
	Veterinarian	$n = 46\ (16.5\%)$	$n = 1\ (1\%)$	$n = 45\ (25.9\%)$
	Rural dweller	$n = 108\ (38.7\%)$	$n = 77\ (73.3\%)$	$n = 31\ (17.8\%)$
Home district	Arpaçay	$n = 26\ (9.3\%)$	$n = 11\ (10.5\%)$	$n = 15\ (8.6\%)$
	Digor	$n = 7\ (2.5\%)$	$n = 0$	$n = 7\ (4\%)$
	Kars central	$n = 193\ (69.2\%)$	$n = 72\ (68.6\%)$	$n = 121\ (69.5\%)$
	Sarıkamış	$n = 24\ (8.6\%)$	$n = 14\ (13.3\%)$	$n = 10\ (5.7\%)$
	Selim	$n = 13\ (4.7\%)$	$n = 2\ (1.9\%)$	$n = 11\ (6.3\%)$
	Susuz	$n = 16\ (5.7\%)$	$n = 6\ (5.7\%)$	$n = 10\ (5.7\%)$
Residence category	City	$n = 110\ (39.4\%)$	$n = 22\ (21\%)$	$n = 88\ (50.6\%)$
	Town	$n = 42\ (15.1\%)$	$n = 6\ (5.7\%)$	$n = 36\ (20.7\%)$
	Village	$n = 127\ (45.5\%)$	$n = 77\ (73.3\%)$	$n = 50\ (28.7\%)$
Site of infection	Face	n/a	$n = 6\ (5.7\%)$	n/a
	Digits	n/a	$n = 33\ (31.4\%)$	n/a
	Hand	n/a	$n = 21\ (20\%)$	n/a
	Wrist	n/a	$n = 15\ (14.3\%)$	n/a
	Arm	n/a	$n = 18\ (17.1\%)$	n/a
	Unspecified cutaneous	n/a	$n = 7\ (6.7\%)$	n/a
	GI tract	n/a	$n = 5\ (4.8\%)$	n/a

Table S2: Substitutions in the dataset

Variable	Missing data type	Number of missing values	Substituted value
Age (y)	Missing data	$n = 2$	$\text{Mean} = 38.69$
Time in occupation (y)	Missing data	$n = 2$	$\text{Mean} = 28.23$
Continuous risk duration (y)	Missing data	$n = 2$	$\text{Mean} = 23.11$
Time since infection (y)	Missing data<br>No recorded infection	$n = 4$<br>$n = 174$	$\text{Mean} = 4.32$<br>$30\ (\max + 5)$
Time since contact (y)	Missing data<br>No recorded contact	$n = 40$<br>$n = 203$	$\text{Mean} = 3.60$<br>$20\ (\max + 5)$

Table S1: Coefficients indicated as likely predictors for anti-PA IgG concentration

Model	Unstandardized Coefficients		Standardized Coefficients	t	Sig. (p)
	B	Std. Error	Beta		
(Constant)	0.843	0.177		4.774	< 0.001
Clinical infection	0.806	0.161	0.376	4.998	< 0.001
Occupation=Veterinarian	−0.485	0.143	−0.173	−3.386	0.001
Occupation=Shepherd	−0.522	0.192	−0.153	−2.724	0.007
Administrative district=ARPAÇAY	0.414	0.182	0.116	2.269	0.024
Log_{10} (Time since infection)	−0.270	0.109	−0.180	−2.487	0.013
Continuous risk duration [y]	0.022	0.010	0.100	2.274	0.024
Occupation=Butcher	0.413	0.142	0.150	2.904	0.004
Residence category=Town	−0.367	0.145	−0.126	−2.526	0.012

Table S2: Coefficients indicated as likely predictors for anti-LF IgG concentration

Model	Unstandardized Coefficients		Standardized Coefficients	t	Sig. (p)
	B	Std. Error	Beta		
(Constant)	−169.059	77.301		−2.187	0.030
Clinical infection	0.641	0.075	0.496	8.563	< 0.001
Residence category=Village	0.257	0.071	0.204	3.624	< 0.001
Administrative district=SARIKAMIŞ	0.455	0.109	0.204	4.166	< 0.001
Sample date	1.25×10^{-8}	0.000	0.112	2.194	0.029
Infection site=Digits	−0.217	0.106	−0.112	−2.047	0.042

Table S3: Coefficients indicated as likely predictors for discriminant score optimised to identify previous infection in individuals with no history of infection

Model	Unstandardized Coefficients		Standardized Coefficients	t	Sig. (p)
	B	Std. Error	Beta		
(Constant)	−0.546	0.062		−8.826	< 0.001
Occupation=Veterinarian	−0.615	0.120	−0.357	−5.131	< 0.001
Continuous risk duration [y]	0.037	0.012	0.219	3.144	0.002

Table S4: Coefficients indicated as least likely predictors for discriminant score optimised to predict infection, using data from individuals with known previous infection

Model	Unstandardized Coefficients		Standardized Coefficients	t	Sig. (p)
	B	Std. Error	Beta		
(Constant)	1.743	0.217		8.046	< 0.001
Occupation=Urban dweller	−0.964	0.351	−0.253	−2.747	0.007
District=Kars central	−0.532	0.249	−0.197	−2.138	0.035
Time since infection [log10 y]	−0.356	0.169	−0.194	−2.104	0.038

Table S7: Characteristics of four individuals with no history of infection, who were identified by discriminant factor analysis as having a >90% probability of having previously had anthrax infection.

Subject	A	B	C	D
Age (y)	29	30	45	74
Gender	Male	Male	Male	Male
Residence category	City	City	Village	Village
Administrative district	Kars Central	Kars Central	Sarıkamış	Kars Central
Occupation group	Butcher	Butcher	Rural dweller	Rural dweller
Time in occupation (y)	18	10	45	74
Continuous risk duration (y)	None	None	Yes, not known	None
Time since contact event (y)	None	None	Yes, not known	5
Anti-PA IgG concentration (µg/ml)	2.7	2.5	1.9	2.9
Anti-LF IgG concentration (µg/ml)	2.0	1.4	2.0	1.5
